# Ligand-receptor interactions combined with histopathology for improved prognostic modeling in HPV-negative head and neck squamous cell carcinoma

**DOI:** 10.1038/s41698-025-00844-6

**Published:** 2025-02-28

**Authors:** Bohai Feng, Di Zhao, Zheng Zhang, Ru Jia, Patrick J. Schuler, Jochen Hess

**Affiliations:** 1 Zhejiang Key Laboratory of Medical Epigenetics, Department of Biochemistry and Molecular Biology, School of Basic Medical Sciences, Hangzhou Normal University, Hangzhou, China; 2 Department of Otorhinolaryngology, Head and Neck Surgery, University Hospital Heidelberg, Heidelberg, Germany; 3 Department of Otorhinolaryngology, Second Affiliated Hospital of Zhejiang University School of Medicine, Hangzhou, China; 4 Department of Pathology, The Second Affiliated Hospital, Zhejiang University School of Medicine, Hangzhou, China; 5 Division Radiooncology/Radiobiology, German Cancer Research Center (DKFZ), Heidelberg, Germany

**Keywords:** Head and neck cancer, Computational biology and bioinformatics

## Abstract

Head and neck squamous cell carcinoma (HNSC) is a prevalent malignancy, with HPV-negative tumors exhibiting aggressive behavior and poor prognosis. Understanding the intricate interactions within the tumor microenvironment (TME) is crucial for improving prognostic models and identifying therapeutic targets. Using BulkSignalR, we identified ligand-receptor interactions in HPV-negative TCGA-HNSC cohort (*n* = 395). A prognostic model incorporating 14 ligand-receptor pairs was developed using random forest survival analysis and LASSO-penalized Cox regression based on overall survival and progression-free interval of HPV-negative tumors from TCGA-HNSC. Multi-omics analysis revealed distinct molecular features between risk groups, including differences in extracellular matrix remodeling, angiogenesis, immune infiltration, and APOBEC enzyme activity. Deep learning-based tissue morphology analysis on HE-stained whole slide images further improved risk stratification, with region selection via Silicon enhancing accuracy. The integration of routine histopathology with deep learning and multi-omics data offers a clinically accessible tool for precise risk stratification, facilitating personalized treatment strategies in HPV-negative HNSC.

## Introduction

Head and neck squamous cell carcinoma (HNSC) represents the sixth most common cancer globally, accounting for approximately 4–5% of all cancer cases, with more than 900,000 new cases diagnosed annually^1,2^. HNSC predominantly affects regions such as the oral cavity, pharynx, and larynx. There is a strong association between HNSC and risk factors such as tobacco use and alcohol consumption^3^. However, over the past few decades, there has been a notable emergence of the human papillomavirus (HPV) as a significant etiological factor, particularly in oropharyngeal cancers^4^. Approximately 25–30% of HNSC cases are HPV-positive, and these cases tend to exhibit superior treatment responses and overall survival rates compared to HPV-negative cases, which are typically more aggressive and associated with inferior clinical outcomes^3,5^. The immune microenvironment in HPV-negative HNSC is frequently characterized by immune evasion mechanisms that foster an immunosuppressive environment, thereby promoting tumor progression and resistance to immune checkpoint inhibitors. It is imperative to gain a deeper understanding of these immune interactions within the tumor microenvironment (TME) in order to develop more effective therapeutic strategies to counter these challenges and improve treatment outcomes^6–8^. This emphasizes the necessity of gaining a deeper understanding of the immune interactions that occur within the TME in order to develop more effective therapeutic strategies.

It is now widely acknowledged that intercellular communication within the TME plays a pivotal role in cancer progression, influencing crucial processes such as tumor growth, metastasis, and immune evasion^9–11^. Ligand-receptor interactions represent a central aspect of this communication, mediating the signaling between cancer cells and the surrounding stromal and immune cells. This creates an intricate network that supports tumor development^12,13^. These signaling pathways have been identified as pivotal regulators of tumor biology, with profound implications for patient prognosis and therapeutic response.

Although high-throughput sequencing and multi-omics technologies have considerably advanced our comprehension of intercellular communication within the TME^14–16^, traditional methodologies that rely exclusively on genomic and transcriptomic data are constrained by certain limitations. The spatial organization of cells, in conjunction with the diverse ligand-receptor signaling networks across different tumor subtypes, introduces further layers of complexity, rendering it challenging to translate molecular insights into clinically actionable models^17,18^. In modern oncology, haematoxylin and eosin (HE) staining remains a cornerstone for solid tumor diagnosis, providing crucial insights into tumor differentiation, budding, and lymphovascular invasion^19,20^. Although HE staining alone does not identify specific immune markers on its own, it provides a foundation for subsequent molecular studies. The incorporation of convolutional neural networks (CNN) has further augmented the capacity to analyze HE-stained slides, thereby enhancing diagnostic precision and addressing the shortage of pathological inspection^21^. Recent developments have facilitated its application to predict genetic alterations^22^, prognosis^23^, HPV status^24^, treatment responses^25^, and microsatellite instability (MSI) ^26^, thereby providing a more profound comprehension of tumor biology.

This study addresses the aforementioned challenge by introducing a novel deep-learning framework that leverages the Silicon pathological region selection strategy to construct a ligand-receptor risk model from HE-stained histopathological slides. This approach integrates spatial information from standard histological techniques to predict the risks associated with ligand-receptor interactions within the TME, thereby offering a more biologically informed and spatially precise prediction of clinical outcomes than is possible with traditional methods based solely on genomic and transcriptomic data.

## Results

### Prognostic risk model based on ligand-receptor pairs for HPV-negative HNSC

**Fig. 1:**
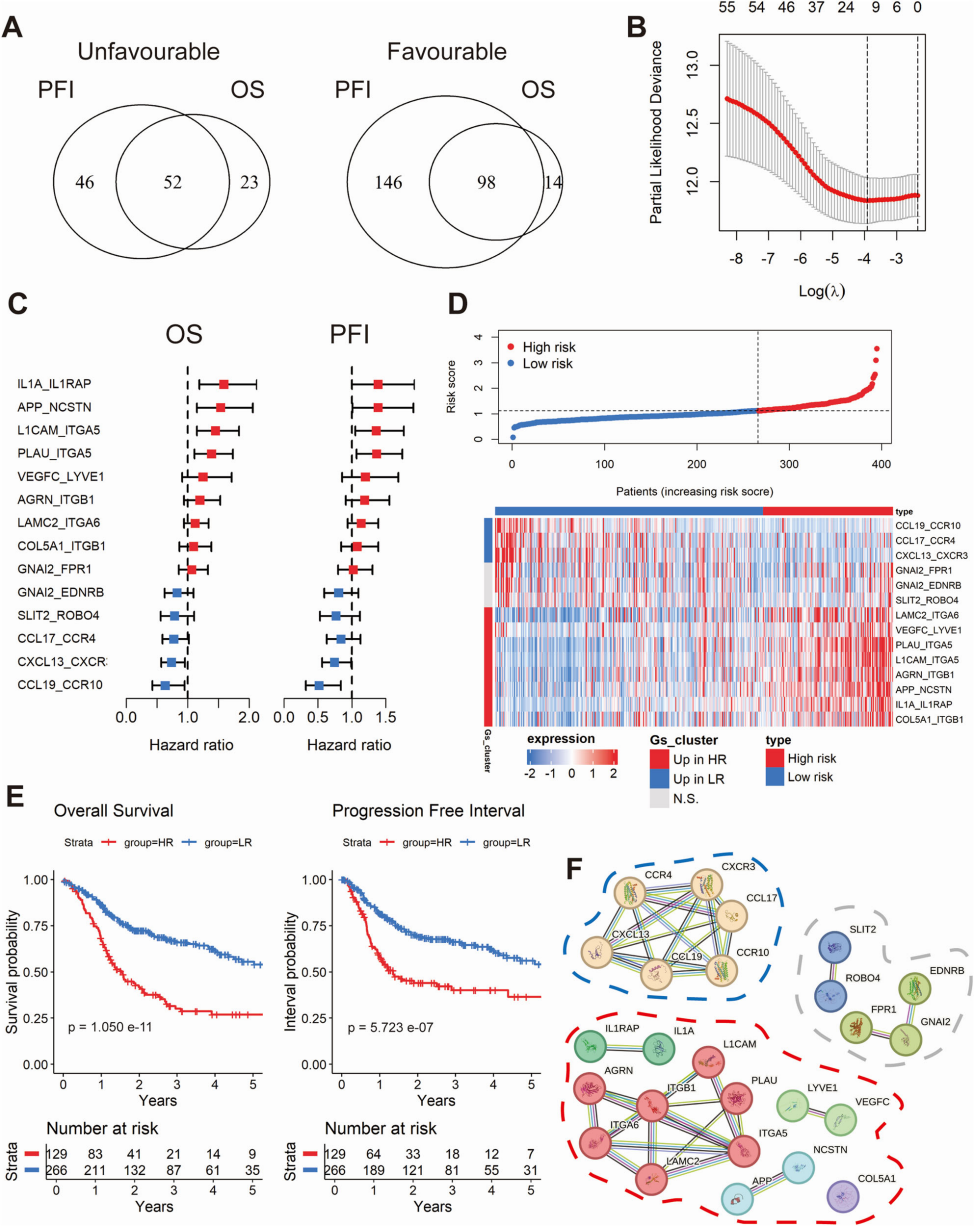
Machine learning-driven establishment of a prognostic model based on ligand-receptor pairs in the TCGA-HNSC-HPV-negative cohort. Venn diagram depicting the overlap between favorable and unfavorable ligand-receptor pairs with significant differences in overall survival (OS) and progression-free interval (PFI), identified using the optimal cut-off method (**A**). Dot plot showing the ligand-receptor pairs most associated with prognosis selected using Lasso Cox modeling based on the minimum partial likelihood deviance (**B**). Forest plots illustrating the univariate regression analysis for OS and PFI across the candidate ligand-receptor pairs (**C**). Dot plot (top) and heatmap (bottom) illustrate the stratification of samples based on the Lasso Cox-derived risk score, segmented by the optimal threshold for OS (**D**). Kaplan-Meier curves showing the prognostic differences in OS and PFI between the stratified sample risk groups (**E**). Network showing the protein-protein interaction (PPI) of genes associated with the ligand-receptor pairs, with clustering performed on the associated genes using k-means (**F**).

The analysis of bulk RNA-seq data using the BulkSignalR package identified 667 ligand-receptor pairs in HPV-negative tumors from TCGA-HNSC (*n* = 395, Supplementary Fig. 1). The best-cutoff algorithm identified a set of 150 pairs that were significantly associated with overall survival (OS) and progression-free interval (PFI), with 52 classified as unfavorable and 98 as favorable (Fig. 1A, Supplementary Tables 1–2). A random forest survival model was employed to refine this set to 55 pairs with importance scores greater than 0.3 (Supplementary Table 1). LASSO-penalized Cox regression was performed to further prioritize 14 ligand-receptor pairs that were most strongly associated with prognosis (Fig. 1B, C). These pairs constituted the basis of a risk model that classified the cohort into two distinct groups: 129 high-risk (HR) and 266 low-risk (LR) tumors, based on an OS cutoff (Fig. 1D). Kaplan-Meier analysis revealed significant differences in OS and PFI between risk groups (Fig. 1E). To further validate the robustness of the risk model, we employed propensity score matching (PSM), adjusting for confounding factors such as tumor size, lymph node metastasis, and radiation therapy. Notably, the differences in OS and PFI between high-risk and low-risk groups remained significant after PSM, underscoring the model’s capacity to provide intrinsic prognostic information independent of tumor stage or treatment modalities (Supplementary Fig. 2A). Furthermore, univariate and multivariate Cox regression analyses for OS again confirmed the model as an independent prognostic factor (Supplementary Fig. 2B, C). A cross-tabulation analysis revealed a significant correlation between high-risk tumors and younger age, perineural invasion (PNI), tumor size, and lymph node metastasis (Supplementary Table 4).

To gain further insight into the molecular characteristics of the ligand-receptor pairs, we constructed a protein-protein interaction (PPI) network and applied k-means clustering to the associated genes (Fig. 1F). In the low-risk group, the majority of pairs were linked to immune cell recruitment, indicating an enhanced immune response. In contrast, high-risk pairs were associated with processes such as extracellular matrix remodeling, angiogenesis, and inflammation, which collectively promote tumor invasion.

### Molecular dynamics and vulnerabilities across prognostic risk groups

**Fig. 2:**
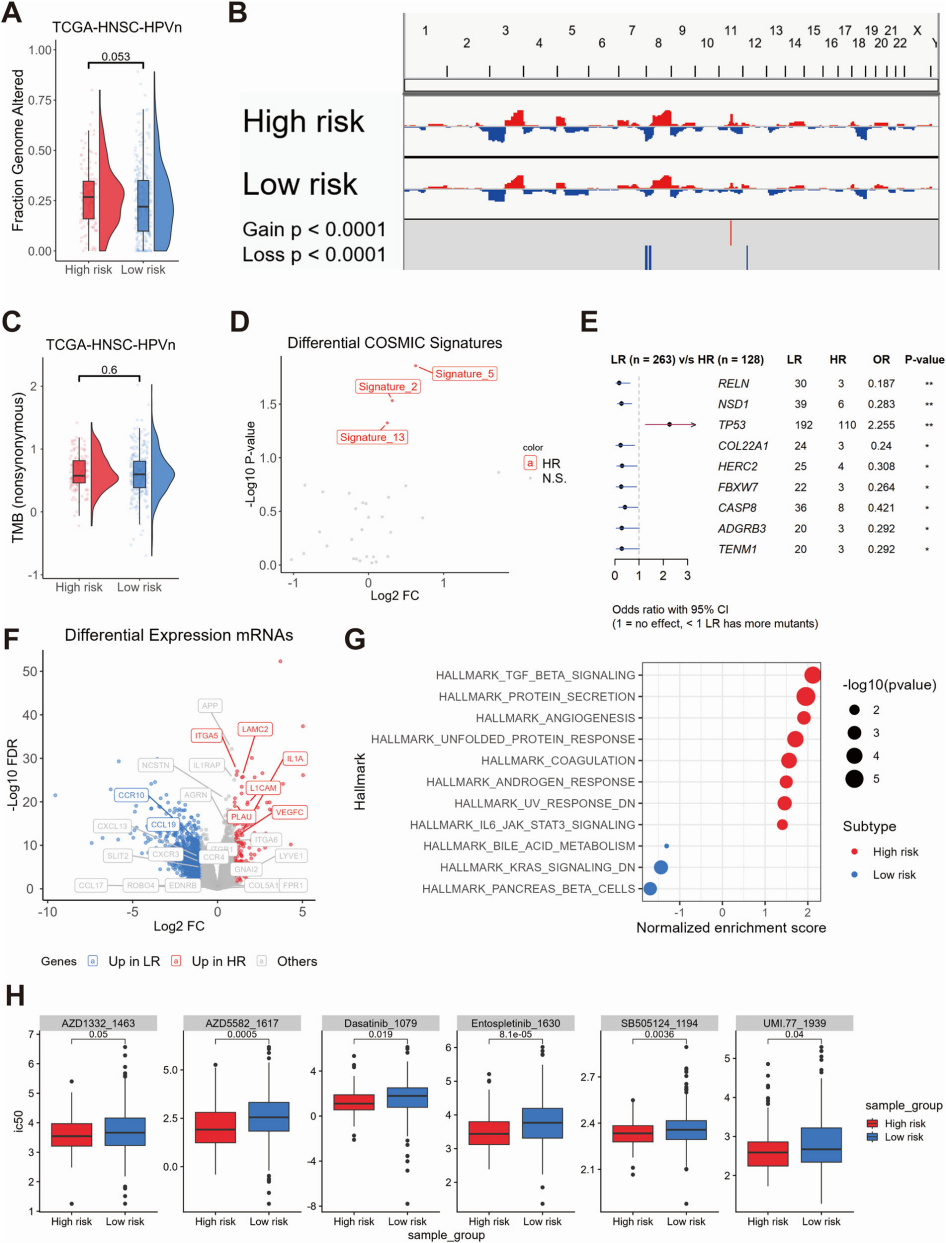
Multi-omics analysis of genomic alterations, gene expression, and drug sensitivity between high-risk and low-risk groups. Combined box and violin plot showing the differences in whole genome alteration levels between the high-risk and low-risk groups (**A**). Copy number variation (CNV) plot illustrating the differences in CNV levels across chromosomes between the high-risk and low-risk groups (**B**). Combined box and violin plot showing the differences in tumor mutational burden (TMB) levels between the high-risk and low-risk groups (**C**). Dot plot illustrating the differences in COSMIC mutational signatures between the high-risk and low-risk groups (**D**). Forest plot showing the genes with statistically significant differences in mutation frequency between the high-risk and low-risk groups **(E)**. Volcano plot displaying differentially expressed genes between the high-risk and low-risk groups, with colors indicating the direction of gene expression changes. Highlighted genes correspond to those included in the ligand-receptor pair risk model (**F**). Dot plot showing the GSEA analysis of Hallmark gene sets between the high-risk and low-risk groups. The size of the dots represents the log10(p-value), and the color indicates the direction of Hallmark gene set enrichment (**G**). Box plot showing the oncopredict-predicted drugs with higher sensitivity in the high-risk group (**H**).

In order to gain insight into the molecular dynamics of different prognostic subtypes, a multi-omics analysis was conducted. A greater number of whole genome alterations were observed in high-risk tumors in comparison to low-risk tumors, although this was not statistically significant (Fig. 2A). Copy number variation (CNV) analysis revealed significant 8p and 12p deletions and 11q amplifications in high-risk tumors (*p* < 0.0001, Fig. 2B), while tumor mutational burden (TMB) showed no significant difference between risk groups (Fig. 2C). However, elevated levels of COSMIC mutational signatures 2, 5 and 13 were observed in high-risk tumors (Fig. 2D). Furthermore, the relative frequency of somatic TP53 mutations was significantly higher for high-risk tumors, whereas the relative frequency of somatic mutations in RELN, NSD1, COL22A1, HERC2, FBXW7, CASP8, ADGRB3 and TENM1 was significantly higher in low-risk tumors (Fig. 2E).

A differential gene expression analysis identified 147 upregulated and 1069 downregulated genes in high-risk tumors (|Log2FC|≥ 1; FDR ≤ 0.05; Supplementary Table 5). These DEGs included several candidates from the ligand-receptor risk model (Fig. 2F). Gene Set Enrichment Analysis (GSEA) demonstrated that high-risk tumors exhibited an enrichment of gene sets associated with extracellular matrix remodeling and the TGF-beta, ECM receptor, integrin, and IL1R signaling pathways (Fig. 2G, Supplementary Tables 6–8). In-silico drug sensitivity analysis using oncoPredict identified six drugs with higher IC50 values in high-risk tumors, including tyrosine kinase inhibitors (Entospletinib, Dasatinib), an IAP antagonist, a TGF-beta/Smad inhibitor, and an MCL-1 inhibitor (Fig. 2H).

### Cellular and spatial distribution of prognosis-related ligand-receptor pairs

**Fig. 3:**
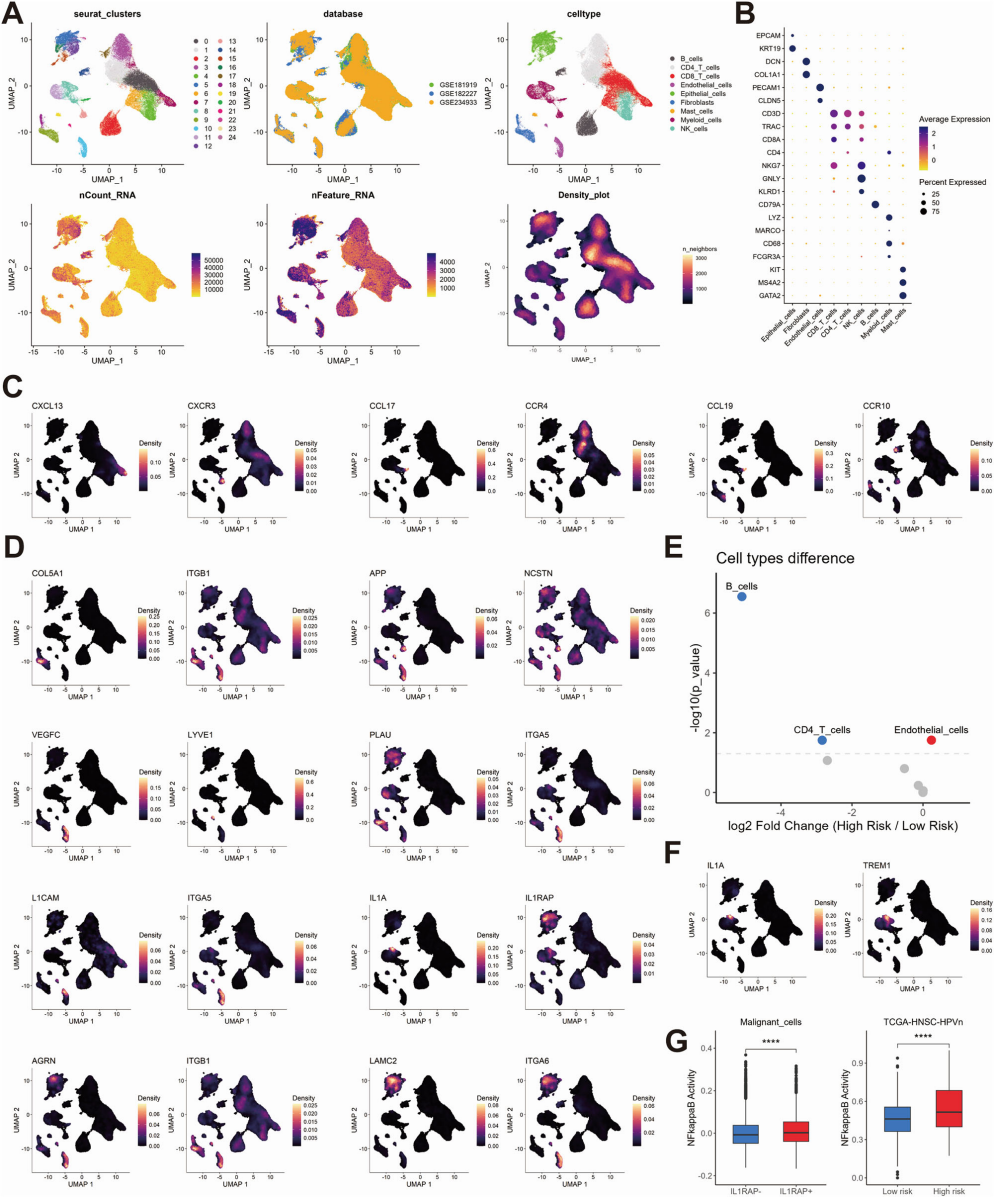
Cellular localization of ligand-receptor pairs and cell component mapping in the TCGA-HNSC-HPV-negative cohort. UMAP clustering plots showing HNSCC cells from the 34 HPV-negative patients across three public databases. Each cell is color-coded for Seurat clusters, databases, cell types, total counts, total features, and cell density (**A**). Dot plot showing the expression of marker genes across different cell types, illustrating the distribution of marker gene expression for each cell type (**B**). UMAP density plots showing the cell-type-specific localization of ligand-receptor pair genes in the low-risk group (**C**) and high-risk group (**D**). Dot plot showing the differences in cellular components between the high-risk and low-risk groups in the TCGA-HNSC-HPV-negative cohort, mapped from single-cell data using the Prism algorithm. Red dots indicate higher abundance in the high-risk group, while blue dots indicate higher abundance in the low-risk group (**E**). UMAP density plots showing the expression distribution of IL1A and TREM1 within myeloid cells (**F**). Box plots showing the differences in NF-kappaB pathway activity between ILRAP1+ and ILRAP1- malignant cells (left) and between the high-risk and low-risk groups in the TCGA-HNSC-HPV-negative cohort (right; (**G**)).

In order to investigate the cellular distribution of prognostic ligand-receptor pairs, we analyzed single-cell RNA-seq data from 34 HPV-negative HNSC samples across three datasets (GSE181919, GSE182227, GSE234933), comprising 115,479 cells after quality control (Fig. 3A, B). Eight major cell types were identified, including epithelial, immune (CD8/CD4 T cells, B cells, mast cells, NK cells, myeloid cells), and stromal cells (fibroblasts, endothelial cells), with significant heterogeneity across samples (Supplementary Fig. 3A, B). A substantial number of CNVs were identified in 14,964 malignant epithelial cells (Supplementary Fig. 3C).

Ligand-receptor pairs that were enriched in low-risk tumors demonstrated interactions between myeloid cells and T and B cells, whereas high-risk pairs indicated interactions between epithelial, fibroblast, and endothelial cells (Fig. 3C, D, Supplementary Fig. 4). The high-risk tumors exhibited a greater abundance of endothelial cell, whereas the low-risk tumors demonstrated a higher prevalence of B and CD4-positive T cells (Fig. 3E). Notably, the expression of IL1A-IL1RAP indicated a specific cellular communication between a subset of myeloid cells and epithelial cells in high-risk tumors. A recent study has demonstrated that IL1 secretion by TREM1-positive myeloid cells within a pro-inflammatory stromal niche can induce treatment resistance in tumor cells via NFkB^27^. Indeed, IL1A expression was most prominent in the subpopulation of TREM1-positive myeloid cells and a significantly higher NFkB activity was evident for IL1RAP-positive malignant epithelial cells (Fig. 3F, G). This finding was further corroborated by a significantly higher NFkB activity single-sample gene set enrichment analysis (ssGSEA) score in HPV-negative tumors with a high-risk phenotype in comparison to their low-risk counterparts from TCGA-HNSC (Fig. 3G).

**Fig. 4:**
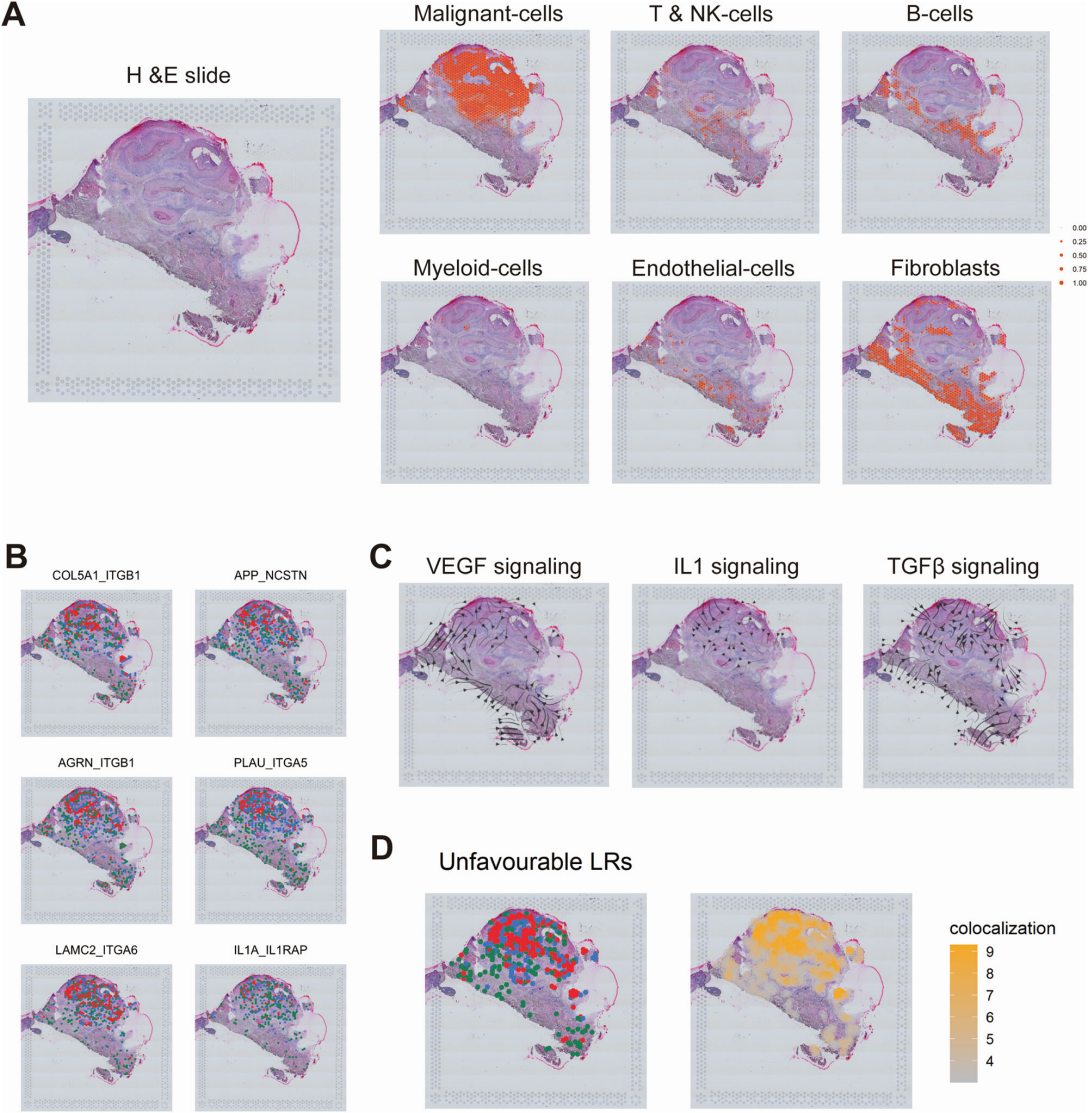
Spatial mapping and colocalization of ligand-receptor pairs and signaling pathways in HNSC tissue. Mapping of different single-cell-derived cell types to tissue spots in HNSC, illustrating the spatial distribution of various cell types within the tissue (**A**). Colocalization of some ligand-receptor pairs within HNSC tissue for high-risk group (**B**). Blue dots represent regions with high ligand expression, green dots represent regions with high receptor expression, and red dots indicate areas where both ligand and receptor are highly expressed. Storm plots showing the distribution and propagation direction of different signaling pathways (VEGF, IL1, TGF-β) within HNSC tissue (**C**). Combined spatial distribution (left) and colocalization scores (right) of eight highly expressed ligand-receptor pairs in the high-risk group within HNSC tissue (**D**).

In order to investigate the spatial distribution of prognostic ligand-receptor pairs, we performed spatial mapping using transcriptomic data (GSM5494476). The cell types identified by scRNA-seq were projected onto tissue spots, thereby revealing the spatial arrangements within the tumor (Fig. 4A). Ligand-receptor pairs linked to high-risk tumors, including IL1A_IL1RAP, COL5A1_ITGB1, APP_NCSTN, PLAU_ITGA5, AGRN_ITGB1, and LAMC2_ITGA6, were found to be particularly enriched in tumor regions (Fig. 4B, S5A-C). Furthermore, storm plots mapped key signaling pathways and demonstrated tumor-stroma interactions via TGF-beta and VEGF, while interaction via IL1 was primarily inferred within the tumor, potentially associated with myeloid cells (Fig. 4C). The co-localization scores revealed the spatial interaction patterns of the high-risk ligand-receptor pairs within the tumor and its surrounding TME (Fig. 4D).

### Classification of high-risk tumors based on histological staining

Following the development of a robust prognostic risk model based on ligand-receptor pairs and an in-depth analysis of their cellular and tissue distribution, our objective was to device a straightforward yet precise classification method utilizing histological HE staining from TCGA-HNSC. Following preprocessing, which included image cropping and color normalization, 3,618,569 individual patches were generated (Supplementary Fig. 6A).

In order to ascertain whether the delineation of pathological regions serves to enhance the performance of deep learning models, we conducted a multi-step analysis. The initial step involved the extraction of deep learning features from all image patches utilizing the ResNet50 model. Subsequently, the feature dimensions were reduced to 32 through the application of principle component analysis (PCA).

**Fig. 5:**
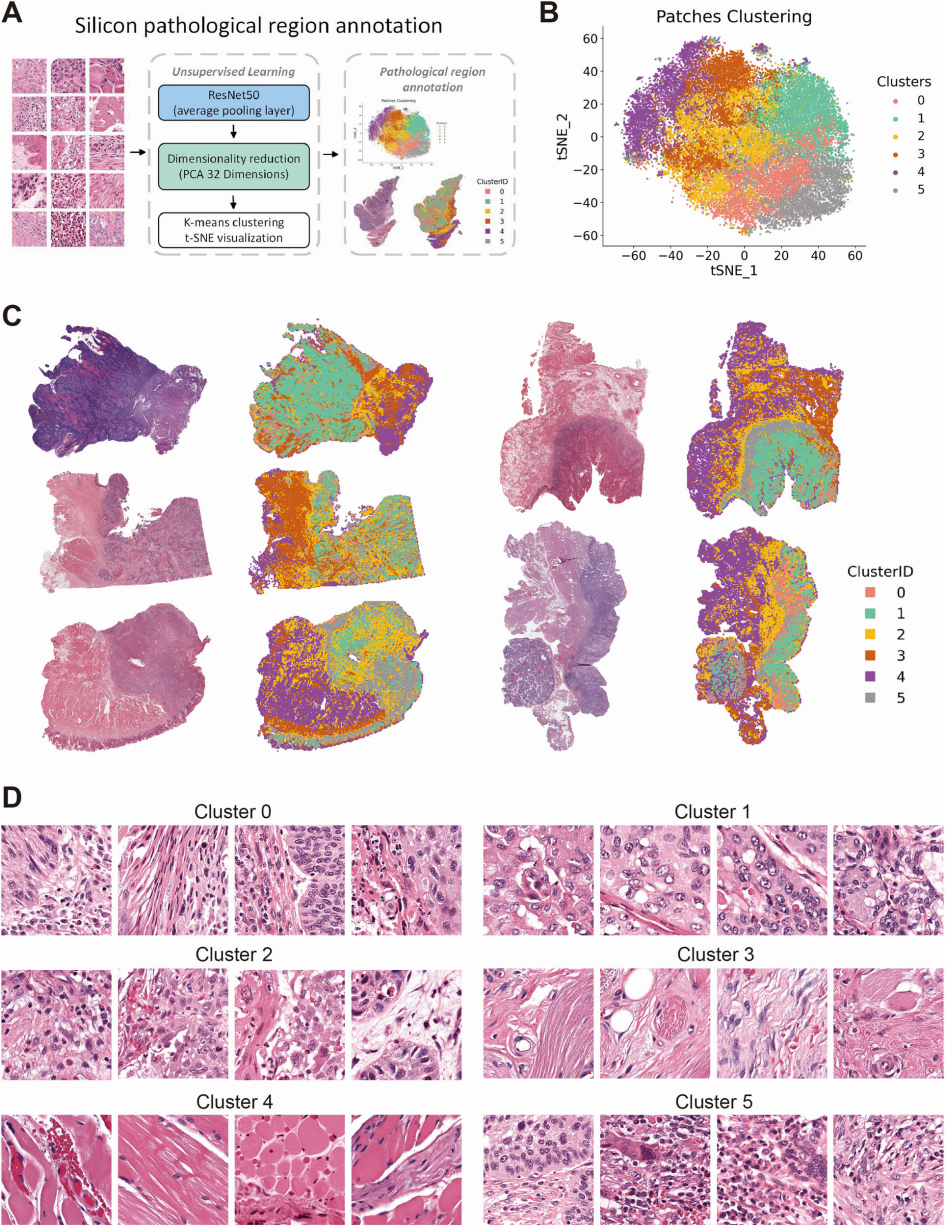
Deep learning-based clustering and spatial visualization of pathological regions in H&E-stained WSIs (whole slide images). Workflow of Silicon pathological region selection. Deep learning features are extracted from patches using a ResNet50 network, followed by dimensionality reduction using Principal Component Analysis (PCA). The reduced features are then clustered using K-means, and the resulting patch clusters are annotated based on histopathological characteristics (**A**). 2D t-distributed Stochastic Neighbor Embedding (t-SNE) visualization of K-means clustering based on deep learning features extracted from patches (**B**). Visualization of the spatial distribution of patches from different clusters on WSI of HE-stained histopathological sections (**C**). Representative images of patches from each cluster (**D**).

Finally, the image patches were clustered into six categories using the K-means clustering algorithm, and resulting clusters were visualized through t-SNE plots (Fig. 5A, B). The clustering results were visualized at the whole slide images (WSIs) level to confirm the distribution of individual clusters for distinct tissue regions (Fig. 5C). An examination of the HE-stained slides revealed that Cluster 0 and 2 were stromal regions in close proximity to tumor tissue, Cluster 1 was identified as tumor tissue, Cluster 3 and 4 were classified as stromal and muscle tissue, and Cluster 5 was determined to be the leading edge of tumor invasion (Fig. 5D).

**Fig. 6:**
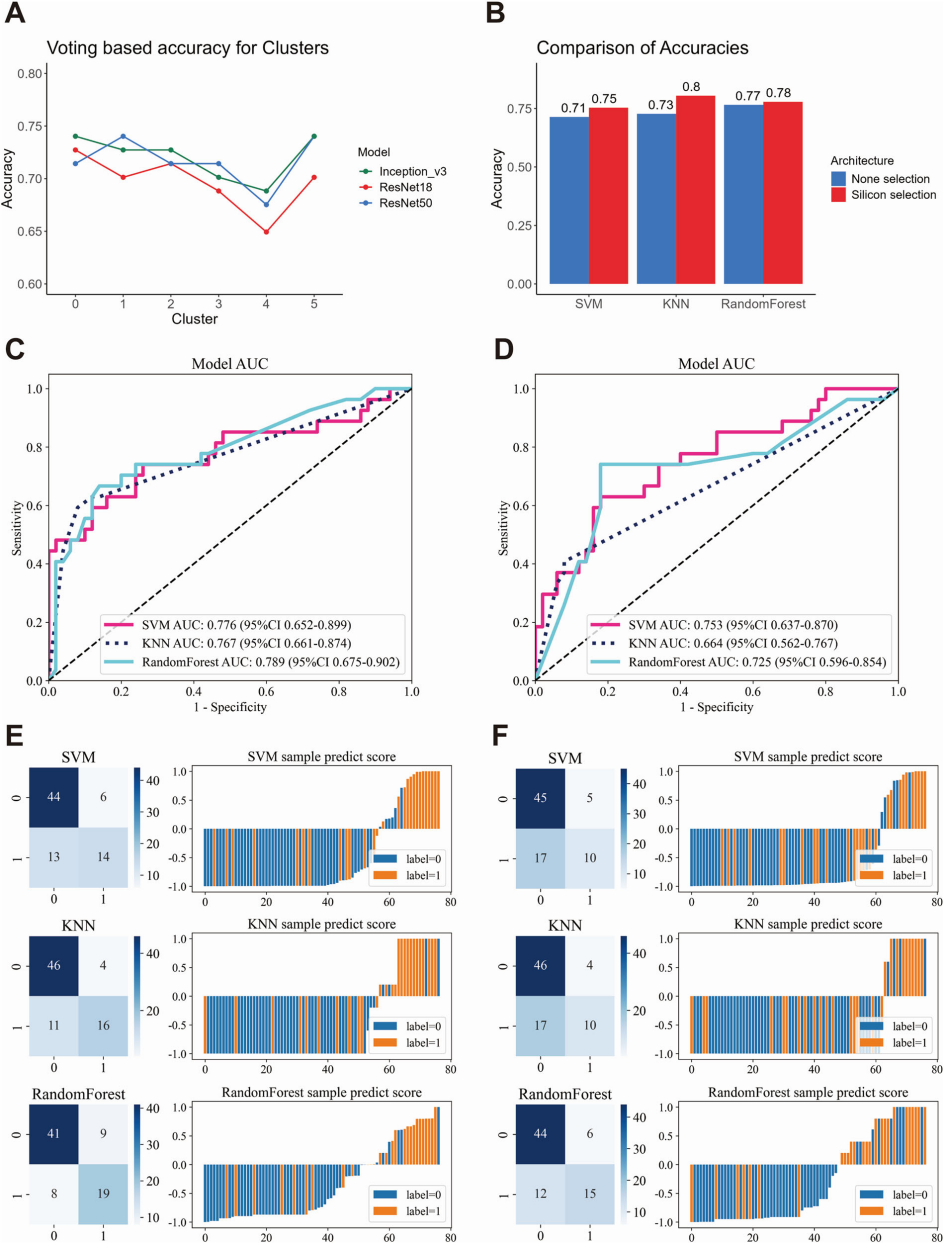
Performance comparison of weakly supervised deep learning models with and without Silicon pathological region selection. Line plot showing the prediction accuracy of weakly supervised learning using different convolutional neural network (CNN) models (Inception_v3, ResNet18, ResNet50) and a voting method across various patch clusters (**A**). Bar plot comparing the accuracy of weakly supervised deep learning models with different machine learning algorithms, contrasting Silicon pathological region selection and non-selection (**B**). Receiver Operating Characteristic curve (ROC) and corresponding Area Under the Curve (AUC) values for multiple machine learning models, comparing the performance of Silicon pathological region selection (**C**) with non-selection (**D**). Confusion matrices and sample prediction score plots illustrate the performance of different machine learning models with deep learning architectures, highlighting the differences between Silicon pathological region selection (**E**) and non-selection (**F**).

Weakly-supervised deep learning was applied using CNN architectures, namely Inception_v3, ResNet18, and ResNet50. The larger networks demonstrated superior performance and identified clusters 3 and 4 (representing stromal and muscle tissue) with reduced accuracy in risk stratification (Fig. 6A). Consequently, we implemented a Silicon pathological region selection process, excluding clusters 3 and 4, with a focus on tumor and adjacent stromal regions. This approach aligned with our findings based on scRNA-seq and spatial transcriptomics data.

### Validation of the Silicon pathological region selection strategy in weakly-supervised deep learning

The objective of this analysis was to ascertain whether the Silicon pathological region selection strategy enhances the efficacy of risk prediction in comparison to weakly-supervised deep learning without selection. A total of 348 cases and 360 HE-stained slides were randomly divided into training and testing cohorts in an 8:2 ratio. Specifically, 275 cases (283 WSIs) were allocated to the training cohort, and 73 cases (77 WSIs) were assigned to the testing cohort. This split was subsequently used for downstream analyses, including development of the risk prediction model (Supplementary Fig. 6B). The CNN Multiple Instance Learning (MIL) architecture (Supplementary Fig. 6C) revealed that models incorporating the Silicon pathological region selection strategy exhibited markedly higher accuracy across diverse machine learning models (Fig. 6B), accompanied by augmented AUC and predictive performance (Fig. 6C–F). Moreover, decision curve analysis (DCA) demonstrated that the model incorporating the Silicon pathological region selection strategy yielded a superior net benefit across a spectrum of threshold probabilities in comparison to the model lacking this selection (Supplementary Fig. 7A-B), substantiating its clinical relevance and potential for augmenting predictive efficacy.

**Fig. 7:**
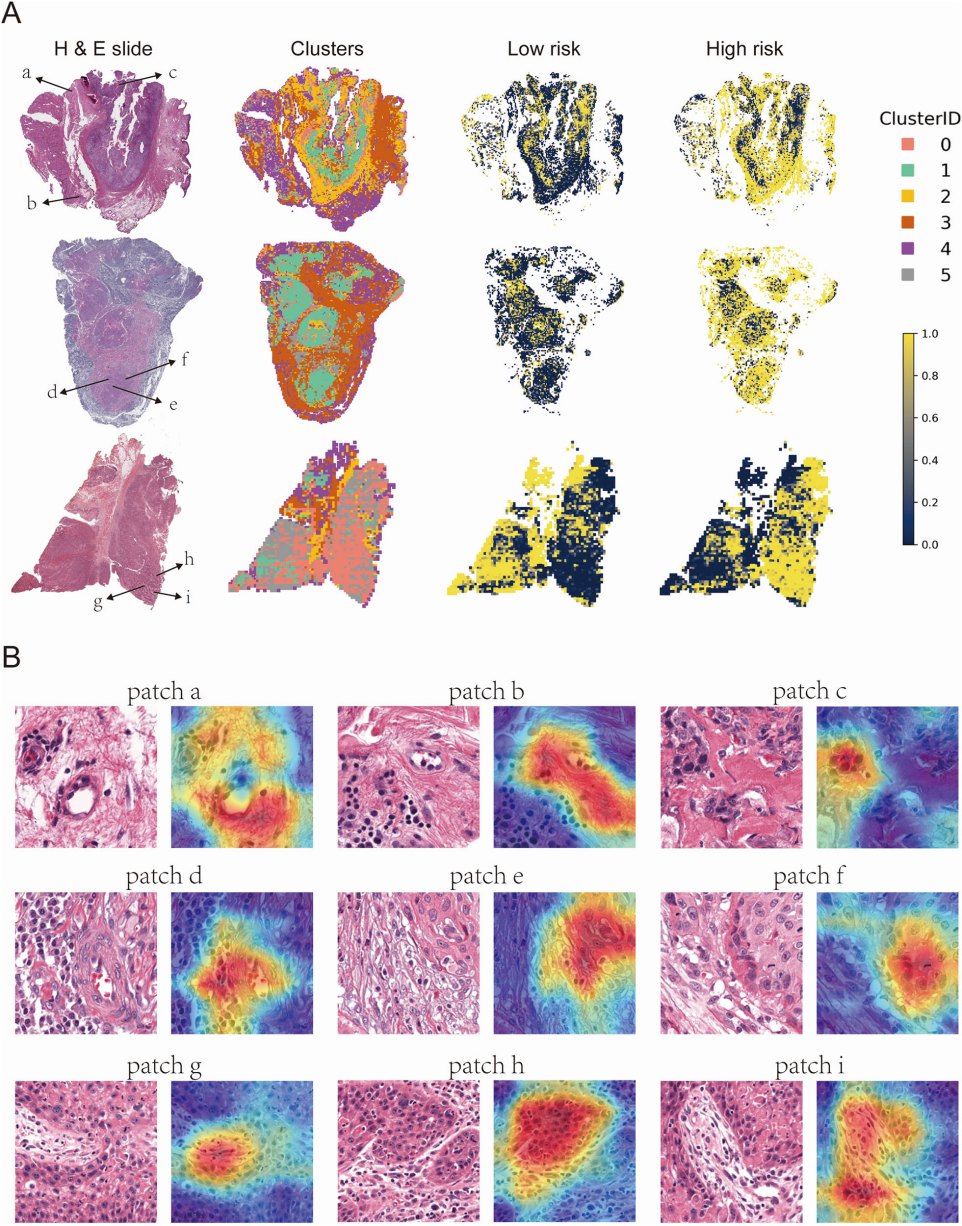
Spatial distribution and representative patches from H&E-stained whole slide images in the high-risk group. Representative WSI images from the high-risk group. The left column shows H&E-stained whole slide images, the second column displays clusters of patches derived from deep learning features, while the third and fourth columns illustrate the spatial distribution of low-risk and high-risk patches, respectively. Clusters are color-coded by Cluster ID (0-5), with yellow representing high-risk areas and dark blue representing low-risk areas (**A**). Representative patches from the H&E-stained slides in (**A**). Each patch (a–i) is annotated with a corresponding Grad-CAM heatmap overlay, highlighting regions of interest identified by deep learning models (**B**).

To gain further insight into the model’s performance, an examination was conducted of the correctly predicted high-risk tumors within the test set (Fig. 7A). In these samples, patches predicted as high-risk exhibited characteristics such as tumor and endothelial cell interaction and tumor-stromal interaction. Grad-CAM heatmaps highlighted high-risk patches concentrated at sites of vascular infiltration and stromal invasion—hallmarks of aggressive tumor phenotypes commonly associated with poor clinical outcomes. This visualization underscores the biological relevance of our model, offering interpretable insights into the key morphological features driving the high-risk classification (Fig. 7B).

**Fig. 8:**
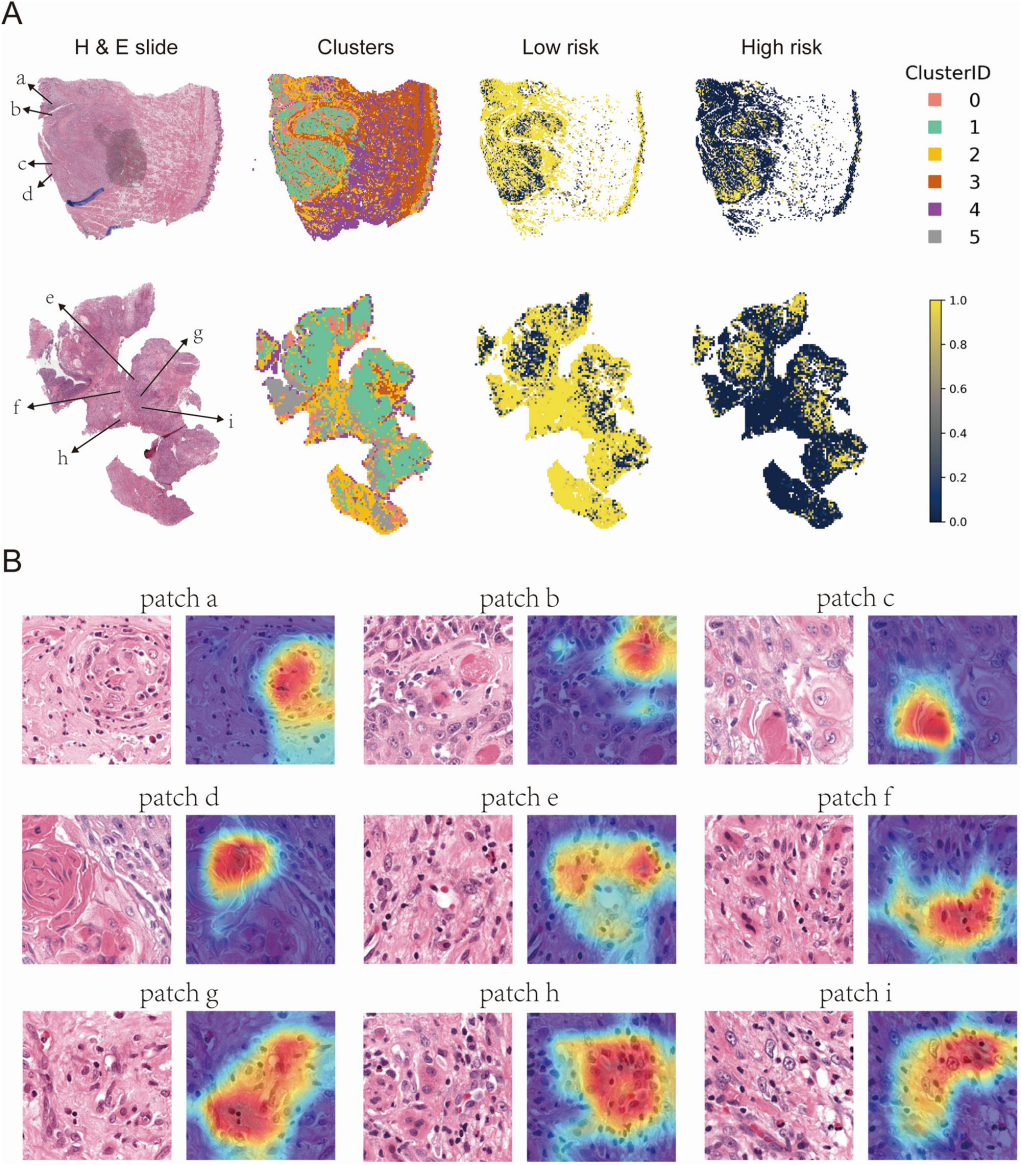
Spatial distribution and representative patches from H&E-stained whole slide images in the low-risk group. Representative WSI images from the low-risk group. The left column shows HE-stained whole slide images, the second column displays clusters of patches derived from deep learning features, while the third and fourth columns illustrate the spatial distribution of low-risk and high-risk patches, respectively. Clusters are color-coded by Cluster ID (0-5), with yellow representing high-risk areas and dark blue representing low-risk areas (**A**). Representative patches from the HE-stained slides in (**A**). Each patch (a–i) is annotated with a corresponding Grad-CAM heatmap overlay, highlighting regions of interest identified by deep learning models (**B**).

Conversely, an examination of the correctly predicted low-risk tumors (Fig. 8A) revealed morphological traits indicative of less aggressive behavior, notably keratinization—a hallmark of well-differentiated tumors—and robust immune cell infiltration, reflecting an active anti-tumor response. Grad-CAM heatmaps highlighted these features (Fig. 8B), both of which have been linked to more favorable clinical outcomes.

## Discussion

In this study, we developed a prognostic risk model for HPV-negative HNSC by integrating multi-omics, scRNA-seq, and spatial transcriptomics data. The identification of key ligand-receptor pairs associated with patient prognosis enabled the establishment of a model that effectively stratified patients into high- and low-risk groups based on OS and PFI. Furthermore, we developed a clinically accessible deep learning model using HE-stained slides, enhanced by the Silicon pathological region selection strategy. Grad-CAM analysis indicated that high-risk tumors were characterized by extracellular matrix remodeling, angiogenesis, and tumor invasion, whereas low-risk tumors exhibited immune cell infiltration and keratinization. This approach illustrates the potential of integrating histopathology with computational methods for accurate clinical prognosis. In the high-risk group, we observed a notable enrichment of COSMIC Signatures 2 and 13, indicative of APOBEC enzyme activity^28^. APOBEC3 enzymes catalyze cytosine deamination, resulting in C to T and C to G mutations. These occur predominantly in single-stranded DNA during the process of replication and repair and have the effect of facilitating substantial genomic alterations that contribute to cancer development and progression ^29–31^. Their overexpression has been demonstrated to exacerbate DNA damage, leading to double-strand breaks and chromosomal rearrangements. This in turn, drives genomic instability, which is a critical feature of many malignancies and fosters tumor heterogeneity and evolution ^29,31^. Furthermore, APOBEC3A contributes to the development of resistance to cancer therapies by accelerating mutational processes that enhance the adaptive potential of cancer cells, particularly in cases of relapse after treatment^32^. Elevated APOBEC activity is strongly associated with a poor prognosis in various cancers, where it drives genomic instability, increasing tumor aggressiveness and recurrence risk^33,34^.

In this study, we observed that the IL1A_IL1RAP signaling pathway was markedly more active in the high-risk group, thereby indicating its contribution to the more invasive characteristics of HPV-negative HNSC. The role of IL-1 signaling in cancer is of great importance, as it has been shown to promote inflammation, tumor growth, and metastasis. It modulates the TME by recruiting immune cells, inducing angiogenesis, and activating pro-inflammatory cytokines^35,36^. Furthermore, studies have shown that IL-1 facilitates angiogenesis by stimulating the production of angiogenic factors such as VEGF and IL-8, thereby promoting tumor growth and metastasis. The increased expression of integrins in response to IL-1 signaling also contributes to the formation of focal adhesions, which stabilize tumor cell attachment to the ECM to enable their invasion^37–39^. More recently, Ji et al. underscored the therapeutic promise of targeting IL-1α to mitigate oxidative stress-induced resistance in oral squamous cell carcinoma (OSCC). Suppression of IL1A expression has been shown to significantly reduce tumor growth and enhance the efficacy of nutrient-starvation therapies, such as Anlotinib. These findings point to the potential of combining IL1A inhibition with current therapeutic approaches to improve clinical outcomes in OSCC^40^. Additionally, Li et al.^27^ emphasized the pivotal function of TREM1-expressing myeloid cells as principal “senders” of IL-1 signals within the TME, exerting a considerable influence on downstream pathways, including the activation of NF-κB in tumor cells. Our findings corroborate this hypothesis, demonstrating elevated NF-κB pathway activity not only in IL1RAP+ malignant cells but also specifically within the high-risk group of HPV-negative HNSC.

On the other hand, our analysis identified ITGB1, ITGA5, and ITGA6 as key integrins significantly associated with poor prognosis in HNSC, underscoring their role in driving tumor aggressiveness and therapeutic resistance. ITGB1, a well-characterized integrin, has been shown to facilitate perineural invasion and radioresistance by modulating cancer stem cell phenotypes, adhesion, and migration in OSCC, thereby contributing to disease progression and therapy failure^41^. ITGA5, frequently overexpressed in invasive tumors, activates PI3K/AKT signaling to promote tumor proliferation and metastasis, marking it as a crucial driver of malignant transformation in HNSC^42,43^. Similarly, ITGA6 exerts its oncogenic effects by engaging the Keap1/Nrf2 and Notch signaling pathways, enhancing oxidative stress resistance and supporting malignant progression^44,45^. Collectively, these integrins orchestrate critical cellular processes, including tumor microenvironment remodeling, cell survival, and metastasis, solidifying their role as pivotal determinants of poor clinical outcomes in HNSC.

Our analysis revealed that patches predicting a high-risk phenotype exhibited distinctive characteristics, including tumor vascular invasion and stromal infiltration. These findings align with the aggressive tumor behavior typically observed in high-risk tumors. Furthermore, the implementation of the Silicon pathological region selection strategy augmented the model’s capacity to accurately predict and classify these pathological characteristics^46^. Conversely, patches classified as low-risk displayed more differentiated features, such as keratin pearls and immune cell infiltration, which are often associated with a more favorable prognoses and less aggressive tumor phenotypes^46,47^. These findings validate the effectiveness of the Silicon pathological region selection strategy, as it enabled a more precise distinction between high- and low-risk patches, thereby enhancing the performance of the deep learning model. Furthermore, our approach employed the patch-based classifier introduced by Wang et al.^48^, which exhibited robust classification capabilities by efficiently identifying pertinent histopathological features and automatically delineating regions of interest (ROI). This not only enhanced the precision of our prognostication but also markedly diminished the requisite manual input from pathologists, thereby optimizing the allocation of both time and resources in clinical settings.

A limitation of this study is the lack of direct experimental validation of the identified high-risk ligand-receptor pairs in tumor samples from independent cohorts. While our analyses provide strong computational evidence, future studies incorporating techniques such as multiplex immunofluorescence are needed to validate these findings and further explore their spatial distribution and biological significance.

This study developed a robust ligand-receptor-based risk model for HPV-negative HNSC by integrating multi-omics, single-cell, and spatial transcriptomics data, thereby uncovering key molecular differences in the tumor microenvironment. Furthermore, we demonstrated the feasibility of applying deep learning to HE-stained slides, enhanced by the Silicon pathological region selection strategy, for accurate risk classification. These findings illustrate the potential of integrating routine histopathology with computational approaches to enhance personalized prognostic assessment in HPV-negative HNSC.

## Methods

### TCGA-HNSC transcriptomic integration

#### Acquisition of TCGA-HNSC information

In November 2021, clinical data and mRNA expression profiles for the TCGA-HNSC cohort was retrieved from the GDC portal (https://portal.gdc.cancer.gov/). Additionally, Tumor Mutation Burden (TMB) and Fraction Genome Altered metrics were sourced from cBioPortal for Cancer Genomics (https://www.cbioportal.org/) during the same period^49,50^. In November 2023, COSMIC mutation data linked to TCGA cohorts were acquired from Zenodo (https://zenodo.org/records/7885656)^51^. Information regarding HPV status was obtained from Cao et al.^52^, where samples with HPV16 expression ≤5 were classified as HPV-negative in HNSC.

### GSEA

GSEA was conducted for Hallmark, Wikipathways, and Kyoto Encyclopedia of Genes and Genomes (KEGG) gene sets, with the normalized enrichment scores and statistical significance calculated. The analysis was performed using the “fgsea” package within the R software environment.

#### ssGSEA

We applied the ssGSEA method from the GSVA package to calculate gene set enrichment scores for the Bulk RNA-seq data, providing insights into pathway activity across samples.

#### The gene signatures collection

Hallmark, Wikipathways, and KEGG gene sets, as well as the BIOCARTA_NFKB_PATHWAY gene set, were obtained using the “msigdbr” package in R.

#### The LASSO Cox analysis

LASSO Cox regression model was applied to identify prognostic markers in HPV-negative HNSC, using the “glmnet” package in the R software environment^53^, and the analytical formula for risk assessment was derived on the basis of 14 prioritized candidate Ligand-Receptor pairs (coefficients: GNAI2_FPR1 = 0.175589783027359; GNAI2_EDNRB = −0.0128431897633999; CCL19_CCR10 = −0.108050441595785; SLIT2_ROBO4 = −0.342190215420395; LAMC2_ITGA6 = −0.0910650517305777; VEGFC_LYVE1 = 0.130137931298295; PLAU_ITGA5 = 0.0431468647033098; L1CAM_ITGA5 = 0.279226243223637; CCL17_CCR4 = −0.0154870550240251; AGRN_ITGB1 = −0.127059688838762; APP_NCSTN = 0.384422861030184; CXCL13_CXCR3 = -0.251637937042456; IL1A_IL1RAP = 0.176155924945072 and COL5A1_ITGB1 = −0.0457893288731351).

#### Survival analysis

Kaplan–Meier survival analysis and univariate Cox regression were performed using the “survival” and “survminer” packages in the R software environment. As TCGA-HNSC tumors with distant metastasis (M1) were excluded from the dataset, the M stage was not considered for the analysis. Optimal cutoff values were determined using the “maxstat” package (smethod = “LogRank”, pmethod = “exactGauss”, abseps=0.001, minprop = 0.3, maxprop=0.7). The propensity score matching analysis was determined using the “MatchIt” package

#### Differential gene expression analysis

Differentially expressed mRNAs between the high and low risk subgroups were identified using the “EdgeR” package in the R software environment^54^.

#### Ligand-receptor level calculation

The ligand-receptor levels in the TCGA-HNSC-HPV-negative cohort were computed using the “BulkSignalR” package^55^.

#### Prognostic importance of ligand-receptor pairs

The prognostic importance of ligand-receptor pairs was assessed using the “randomForestSRC” and “randomSurvivalForest” packages, with parameters set to ntree = 1000 and seed = 666^56^.

#### Somatic mutation analysis

In January 2022, somatic mutation data for the TCGA-HNSC cohort were retrieved using the “TCGAbiolinks” package in the R software. The subsequent somatic mutation analysis was conducted with the “maftools” package within the R environment^57^.

#### CNV analysis

In January 2022, TCGA copy-number variation data were retrieved using the “TCGAbiolinks” package in R. Segment_Mean values greater than 0.3 were classified as gains, while values less than −0.3 were considered losses. Statistical analysis was performed using the Fisher’s exact test implemented in the “CoNVaQ” R package^58^. CNV summary plots were generated with IGV version 2.7.2^59^.

#### Drug resistant scoring

Drug resistant scores for the TCGA-HNSC cohort were calculated using the “oncoPredict” package^60^.

### Single-cell transcriptomic integration

#### Single-cell dataset acquisition

In September 2023, We retrieved three single-cell RNA sequencing datasets (GSE181919^61^, GSE182227^62^, and GSE234933^63^) from the NCBI Gene Expression Omnibus (GEO) and processed them into Seurat objects using the Seurat v4.4.0 R package^64^ for downstream analysis. Clinical data, including HPV status, were also obtained from the GEO.

#### Single-cell data quality control

To ensure data quality, we applied stringent filtering criteria. For both GSE181919 and GSE182227, cells were retained if they had between 250 and 5000 detected features, less than 10% mitochondrial gene expression, less than 1% hemoglobin gene expression, more than 10% ribosomal gene expression, and over 1000 total RNA counts. For GSE234933, the same criteria were used, except cells with more than 2500 detected features were excluded.

#### Normalization and feature selection

We performed data normalization and feature selection on the single-cell RNA-seq data using the SCTransform function, limiting variable features to the top 5000 genes. After identifying these features, we refined the feature list by excluding certain gene sets, such as stress-related genes, long intergenic non-coding RNAs (LINC), mitochondrial genes, immunoglobulin genes, and other unwanted gene categories, including those beginning with “MT-“, “RP[SL]”, and “TR[ABDG]”. Additionally, we filtered out genes with specific suffixes such as “-AS”, “-DT”, and those representing pseudogenes or certain immunoglobulin variants (e.g., IGHV, IGLV)^65^.

#### Dimensionality reduction and clustering

After refining the feature list, we conducted principal component analysis (PCA) using the RunPCA function in Seurat. To reduce dimensionality and cluster the single-cell RNA-seq data, we first determined the optimal number of principal components (PCs), selecting the top 27 PCs that accounted for over 85% of the variance. To correct for batch effects, we applied the Harmony algorithm^66^, followed by UMAP for visualization. We then identified cell neighborhoods and clusters using the FindNeighbors and FindClusters functions, with a resolution of 0.5. Clusters were assigned as cell identities, and cluster levels were standardized from 0 to 24 for consistency in downstream analysis. Finally, cell clusters were annotated based on known biological cell types using canonical marker genes.

#### Single-cell CNV analysis

We used the inferCNV package to detect CNVs and distinguish malignant cells. Afterward, cells were grouped via k-means clustering, and epithelial cells with abnormal CNV profiles were identified as potential malignant cells.

#### Single-cell gene set enrichment score

We calculated single-cell gene set enrichment scores using the AddModuleScore function, allowing for the assessment of gene activity at the individual cell level.

#### Integration of single-cell and bulk RNA-seq data

We integrated the single-cell RNA-seq data with bulk RNA-seq data and performed correlation and outlier analysis using the BayesPrism package^67^. After filtering and cleaning the single-cell data, we selected protein-coding genes and conducted differential expression analysis. Finally, we applied BayesPrism to deconvolute the bulk RNA-seq data, estimating the proportions of different cell types within the bulk samples.

### Spatial transcriptomic integration

#### Spatial transcriptomics dataset acquisition

In November 2022, the spatial transcriptomics dataset GSM5494476 in GSE181300^68^ was obtained from the NCBI GEO for subsequent analysis. We first loaded the feature-barcode matrix and corresponding high-resolution tissue image using the Load10X_Spatial function. The data were normalized using SCTransform, retaining the top 3000 variable features, followed by PCA to reduce dimensionality.

#### Ligand-receptor enrichment analysis

We conducted ligand-receptor enrichment analysis on spatial transcriptomics data using the Seurat package. Ligand and receptor expression levels were extracted and summed across spatial coordinates. Expression values were normalized and log-transformed to account for variability across samples. Cells were categorized as ligand_high, receptor_high, or BothHigh based on their expression levels relative to the top 20% of cells. Specifically, for each ligand or receptor, cells with expression levels in the top 20% were designated as ligand_high or receptor_high. Cells that exhibited both ligand and receptor expression in the top 20% were classified as BothHigh. This approach highlights the cells with the highest expression of ligands and receptors, emphasizing regions of potential signaling activity.

#### Ligand-receptor colocalization analysis

For ligand-receptor colocalization analysis, we estimated interaction strength between ligands and receptors based on spatial proximity. Using a nearest-neighbor algorithm (knn=6), we identified the six nearest neighboring cells for each cell based on spatial coordinates. Two interaction scores were then calculated: one by multiplying the ligand expression of a cell with the maximum receptor expression among its neighbors, and the other by multiplying the receptor expression of a cell with the maximum ligand expression among its neighbors. The higher of the two values was selected as the final interaction score for each cell. To normalize the data, interaction scores were log-transformed, and to control for outliers, the scores were capped at the 95th percentile. The spatial patterns of ligand-receptor colocalization were visualized using ggplot2, with color gradients representing the strength of these interactions across the tissue.

#### Commot-based spatial communication analysis

We analyzed the spatial transcriptomics dataset GSM5494476 using Scanpy^69^ and Commot^70^. The dataset was normalized, log-transformed, and filtered to select highly variable genes. Cell-cell communication was assessed using the CellChat^71^ ligand-receptor interaction database, with interactions filtered based on a minimum expression threshold of 5% of cells. Spatial communication analyses were performed with a 500-micron distance threshold, and communication patterns were visualized through stream plots.

#### Integration of single-cell and spatial data

Anchors were identified between the single-cell reference (scRNA-seq) and the Spatial Transcriptomic data using FindTransferAnchors in the Seurat v4.4.0 R package^64^, which facilitates the integration of the two datasets. These anchors were used with the Seurat TransferData function to map cell-type identities from scRNA-seq to the spatial data.

#### Protein-protein interaction analysis

Protein-protein interaction analysis was performed using the STRING online database, and interactions were clustered into 8 groups using the built-in k-means (obsolete) functionality.

### Methodology for ligand-receptor risk model construction using HE slide image

#### Acquisition and preprocessing of WSIs

We acquired a total of 348 HPV-negative HNSC cases from the GDC portal (https://portal.gdc.cancer.gov/), comprising 360 diagnostics HE-stained slide images (12 with two slides; 47 with no slides). To effectively manage the extensive collection of digitally processed images, we employed a systematic preprocessing strategy. This involved segmenting WSIs into 512 × 512 pixel tiles magnified 20 times, prioritizing high-quality data acquisition. To this end, we eliminated white backgrounds based on color saturation, utilizing a brightness threshold of 216, resulting in a total of 3,618,569 patches.

A total of 348 cases, along with 360 HE-stained slides, were randomly assigned to training and testing cohorts. The training cohort consisted of 275 cases (283 WSIs, 2,833,360 patches), while the testing cohort included 73 cases (77 WSIs, 785,209 patches). To ensure reproducibility in the random assignment, the seed value was set to 666. This 8:2 division was implemented to facilitate downstream analyses, including the development of the risk prediction model.

#### Deep learning training

After the patches were normalized using the Vahadane method for color correction and Z-score normalization of the RGB channels to standardize intensity values, we utilized the CNN architecture for patch-level classification. For the deep learning model, some architectures (ResNet-18, ResNet-50, and Inception_v3) were selected for patch-level classification. To enhance model performance, transfer learning was applied by initializing the model with weights pre-trained on the ImageNet dataset. This approach allowed the model to leverage pre-learned features while being fine-tuned on our dataset, which consisted of weakly annotated patches extracted from the WSIs. During training, we employed stochastic gradient descent (SGD) as the optimization method. The learning rate was dynamically adjusted using a cosine decay learning rate scheduler, with the initial learning rate set at 0.01. This approach ensured a gradual reduction in learning rate, optimizing convergence during the training process. To further improve the model’s ability to generalize across diverse tissue structures and staining variations, data augmentation techniques were applied. These included random horizontal and vertical flipping of the patches during the training phase. Only normalization was applied during the testing phase to ensure consistency and prevent data leakage.

#### Majority voting for cluster model screening

During the coarse screening of different clusters, we employed a majority voting approach to aggregate patch-level predictions into clustered WSI-level classifications. Each clustered WSI was divided into non-overlapping patches, and predictions were generated for each patch by the deep learning model. If more than 50% of the patches in a clustered WSI were classified as 1, the clustered WSI was labeled as 1; otherwise, it was labeled as 0. This method enabled efficient evaluation of the models across clusters by summarizing the dominant prediction from patch-level outputs, facilitating the selection of high-performing clusters.

#### Patch clustering

Morphological features were extracted from each patch using deep learning techniques, facilitating the comparison of similarities and differences among the patches. The patches were clustered in the feature domain to enable this analysis, providing insights into their morphological distribution and potential clinical implications. Using ResNet-50, we extracted 2048 deep features from the averaging pooling layer for each patch. We then applied PCA clustering to compress these 2048 deep features into 32 principal component features. Utilizing the extracted features, we implemented a K-means clustering algorithm to partition the patches into k clusters. The optimal value of k was set to 6, informed by pathologists’ interpretations of the patch clustering in relation to the WSIs, alongside the pathological characteristics and organizational distribution patterns of each patch. The patches in different clusters were considered to exhibit discriminative imaging patterns related to tumor pathological features.

#### Silicon pathological region selection strategy

Next, 3 separate patch-level CNN classifiers were trained on the 6 patch clusters for all patients in the training cohort, respectively, where the ResNet-18, ResNet-50, and Inception_v3 were used as the CNN architecture (parameters: batch size = 128, epochs = 50). The 6 clusters obtained in the validation cohort were optimized for their corresponding classifiers. We recorded the accuracy of each cluster using a voting-based approach. Additionally, we incorporated pathologists’ insights into the characteristics of each patch type, including cell types related to ligand-receptor interactions, histopathological distribution, and the performance of each cluster across different models. By integrating these three factors, we selected the most relevant clusters for further analysis.

#### Performance testing of silicon pathological region selection

We evaluated the performance of the Silicon Pathological Region Selection Strategy by integrating clusters of patches selected using this method for electronic annotation. ResNet-50 was applied for weakly supervised deep learning (parameters: batch size = 128, epochs = 50) on the annotated patch regions, and the same process was conducted on all patches (without applying the selection strategy). The effectiveness of the strategy was assessed by comparing accuracy, ROC AUC scores, and confusion matrices across Strategies.

#### Multi-instance learning for WSI fusion

Following patch-level classification, we applied multi-instance learning to aggregate patch-level predictions into WSI-level predictions. Two approaches were utilized for this purpose: the Patch Likelihood Histogram (PLH) and Bag of Words (BoW) pipelines. In the PLH approach, patch likelihoods were discretized and summarized into histograms, capturing the distribution of patch predictions across the WSI. The BoW approach, on the other hand, employed a term frequency-inverse document frequency (TF-IDF) mapping for each patch, transforming the likelihoods into feature vectors that were used for training machine learning classifiers. These classifiers aggregated patch-level information to produce a single prediction for each WSI. This multi-instance learning approach enabled the model to integrate patch-level information and generate WSI-level predictions, improving stratify accuracy by considering the spatial distribution of relevant features across the entire slide.

#### Machine learning signature building and evaluation

After obtaining the data from the PLH and BoW pipelines, we performed feature selection based on Pearson correlation coefficients. Features with high correlation were considered redundant, and only one was retained to avoid multicollinearity. Specifically, we used a threshold of 0.8 to identify and filter out highly correlated features, reducing the feature space to the top 32 features. Then, we employed three machine learning algorithms—Support Vector Machines (SVM), k-Nearest Neighbors (KNN), and Random Forests—for this purpose. These selected feature vectors were then used to train the SVM, KNN, and Random Forest models. SVM used the radial basis function (RBF) kernel to capture non-linear relationships in the data, while Random Forests combined predictions from multiple decision trees to enhance model robustness. KNN was included as a non-parametric classifier that relied on proximity between data points for classification.

To assess the performance of our models, we used a comprehensive evaluation approach. The primary metric for evaluating the classification accuracy was the area under the receiver operating characteristic curve (AUC), both at the patch level and the WSI level. For patch-level predictions, we generated receiver operating characteristic (ROC) curves to visualize and compare model performance across different classifiers. At the WSI level, patch predictions were aggregated, and the resulting WSI predictions were also evaluated using AUC as the main metric. Additionally, we employed confusion matrices to further analyze model performance, providing a clear visualization of true positives, false positives, true negatives, and false negatives. This allowed us to assess model sensitivity and specificity in greater detail.

All of these models were implemented using scikit-learn, a widely used machine learning library in Python data science.

#### Patch and WSI visualization

Patch visualization was performed using Grad-CAM to generate heatmaps highlighting regions contributing to the model’s predictions. For WSI visualization, patch predictions were mapped back to their original coordinates, with visualizations created using Matplotlib to show the spatial distribution of model outputs across the WSI.

### Software and statistics

The study employed a range of software tools, including custom Python code written in Python v.3.7.12. The Python packages used in the analysis included Pandas v.1.2.4, NumPy v.1.20.2, PyTorch v.1.8.0, OpenSlide v.1.2.0, Seaborn v.0.11.1, Matplotlib v.3.4.2, SciPy v.1.7.3, and scikit-learn v.1.0.2.

Additionally, custom R code was written in R v.4.4.0, utilizing various R packages such as Seurat v.4.4.0, harmony v.1.2.0, msigdbr v.7.5.1, GSVA v.1.52.0, fgsea v.1.30.0, clusterProfiler v.4.12.0, ggplot2 v.3.5.1, infercnv v.1.20.0, BayesPrism v.2.2.2, BulkSignalR v.0.0.9, oncopredict v.1.2, maftools v.2.20.0, maxstat v.0.7-25, TCGAbiolinks v.2.32.0, survminer v.0.4.9, oncoPredict v.1.2, randomForestSRC v.3.3.1, and randomSurvivalForest v.3.6.4, all run within the R 4.4.0 environment. The convaq v.0.1.3 package was run separately within the R 3.6.3 environment.

## Supplementary information

Supplementary information

Supplementary information

## Data Availability

All datasets used in this study are publicly available from their respective sources. TCGA-HNSC Data: Clinical data, mRNA expression profiles, and WSIs for the TCGA-HNSC cohort were retrieved from the GDC portal (https://portal.gdc.cancer.gov/). TMB and Fraction Genome Altered metrics were obtained from cBioPortal (https://www.cbioportal.org/). Mutation Data: COSMIC mutation data linked to TCGA cohorts were acquired from Zenodo (https://zenodo.org/records/7885656). Somatic mutation and CNV data were retrieved using the "TCGAbiolinks" R package. Single-cell RNA-seq Data: Single-cell transcriptomic datasets (GSE181919, GSE182227, and GSE234933) were obtained from the NCBI Gene Expression Omnibus (GEO) (https://www.ncbi.nlm.nih.gov/geo/). Spatial Transcriptomic Data: The spatial transcriptomics dataset GSM5494476 (GSE181300) was retrieved from the NCBI GEO database.

## Code availability

Some or all of the data, models, and code used or generated in this study are available upon request from the corresponding author.
