# Supplementary material for: Ligand-receptor interactions combined with histopathology for improved prognostic modeling in HPV-negative head and neck squamous cell carcinoma
Source: NPJ Precis Oncol. 2025 Feb 28;9:57. doi: 10.1038/s41698-025-00844-6 (PMC11871237; doi:10.1038/s41698-025-00844-6)
Supplement: Supplementary file 1 — Supplementary information [file 41698_2025_844_MOESM1_ESM.pdf]

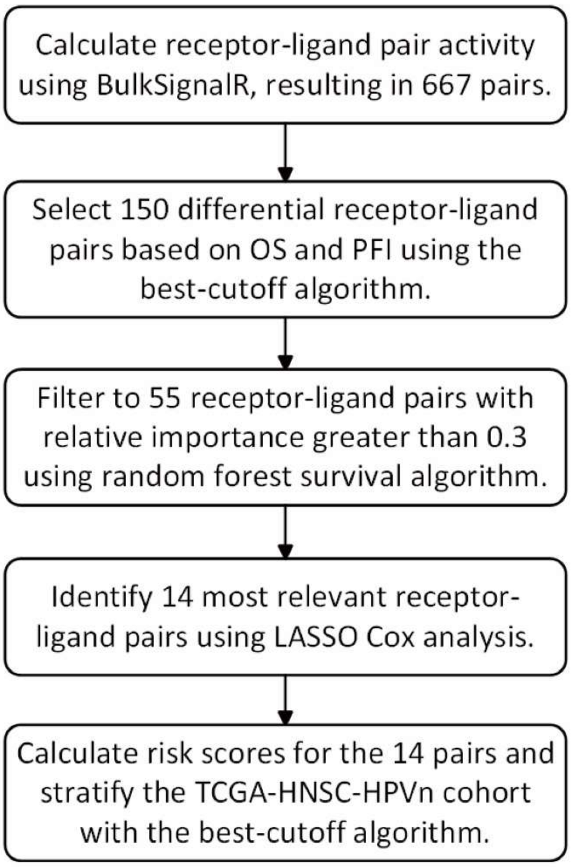

Supplementary Figure 1. Workflow for selecting receptor-ligand pairs.

The flowchart illustrates the process of selecting receptor-ligand pairs, starting with BulkSignalR to calculate activity for 667 pairs. The selection was refined using the best-cutoff algorithm, random forest survival, and LASSO Cox analysis, resulting in 14 pairs used for risk score calculation and cohort stratification.

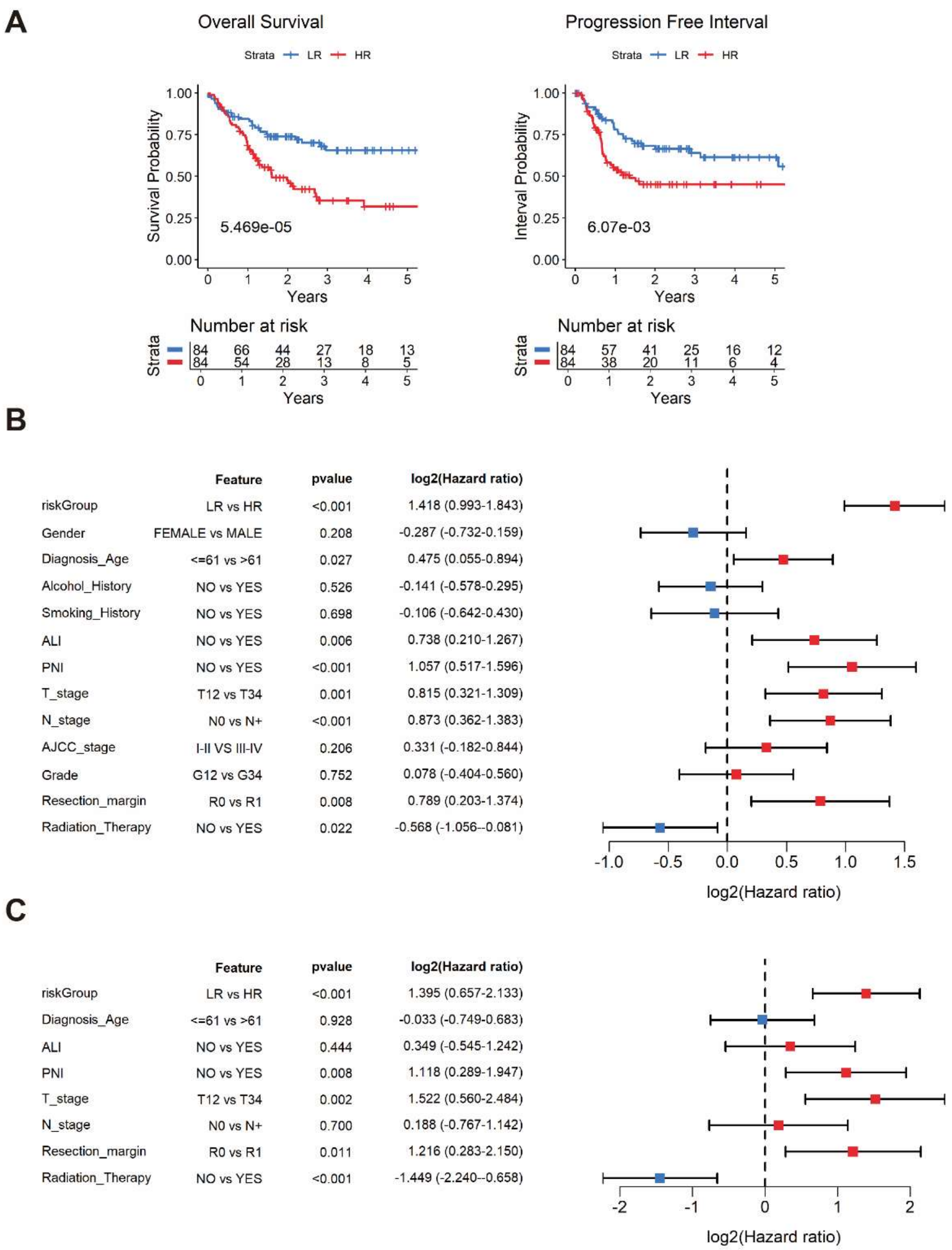

Supplementary Figure 2. Univariate and multivariate Cox regression analysis of risk score and other clinical factors.

Kaplan-Meier survival analysis for overall survival (OS) and progression-free interval (PFI) in high-risk and low-risk groups after propensity score matching (PSM) adjusted for tumor size, lymph node metastasis, and radiation therapy **(A)**.Univariate Cox regression analyses of risk score and other clinical factors. The forest plots display the hazard ratios (HRs) with 95% confidence intervals (CIs) for each variable, including risk score, gender, diagnosis age, alcohol history, smoking history, ANI,

PNI, T stage, N stage, AJCC stage, grade, resection margin and radiation therapy. M stage was excluded from the analysis due to the absence of M1 samples **(B)**. Multivariate Cox Regression Analyses of risk score and the significant clinical factors identified in the Univariate Cox regression analyses. Significant associations ($p < 0.05$) highlight the independent prognostic value of the risk score and other clinical factors **(C)**. Both in univariate and multivariate Cox regression plot the information listed to the left of the "Feature" column serves as the reference group for the corresponding variables.

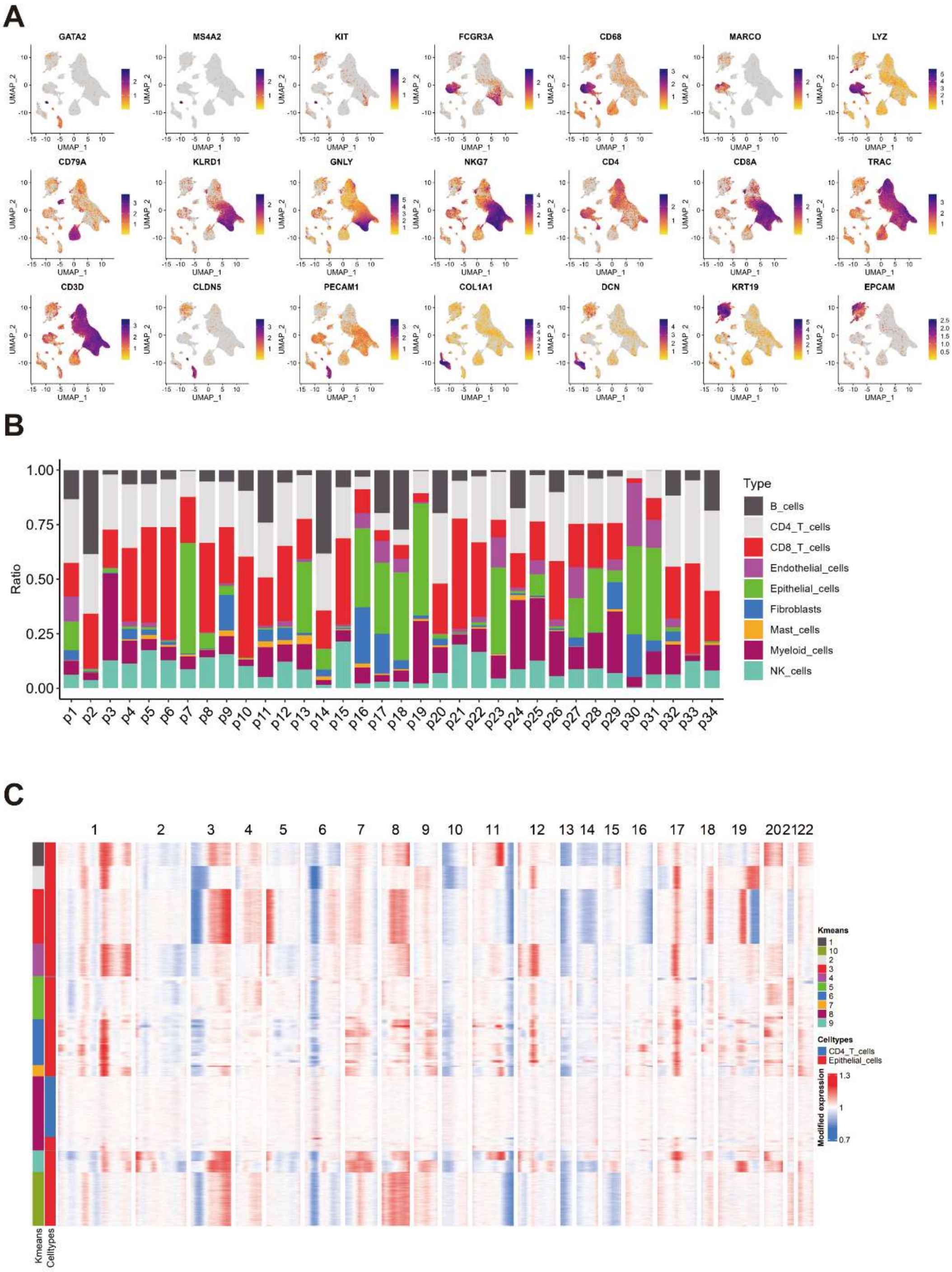

Supplementary Figure 3. Cell marker expression and compositional analysis in single-cell data.

UMAP plots showing the expression of various marker genes across different cell types in single-cell data **(A)**. Stacked bar plot illustrating the percentage distribution of various cell types across different samples **(B)**. Heatmap of K-means clustering for CNV in epithelial cells, using CD4 T cells as a negative reference **(C)**.

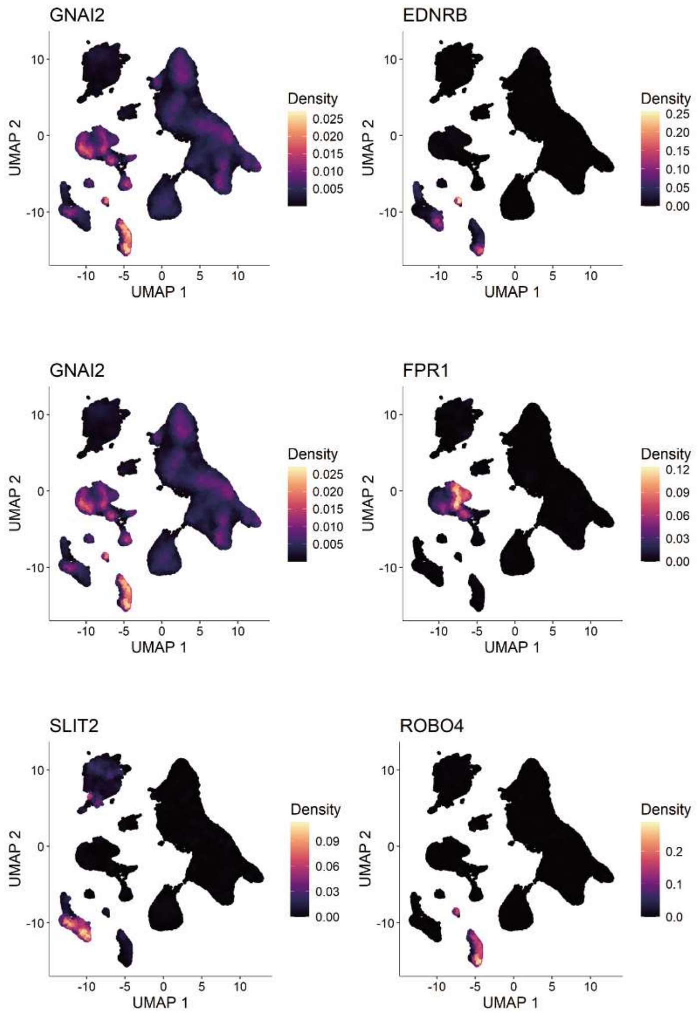

Supplementary Figure 4. Cellular localization of non-significant ligand-receptor pairs

UMAP density plots showing the cell-type-specific localization of ligand-receptor pair genes in the non-significant group.

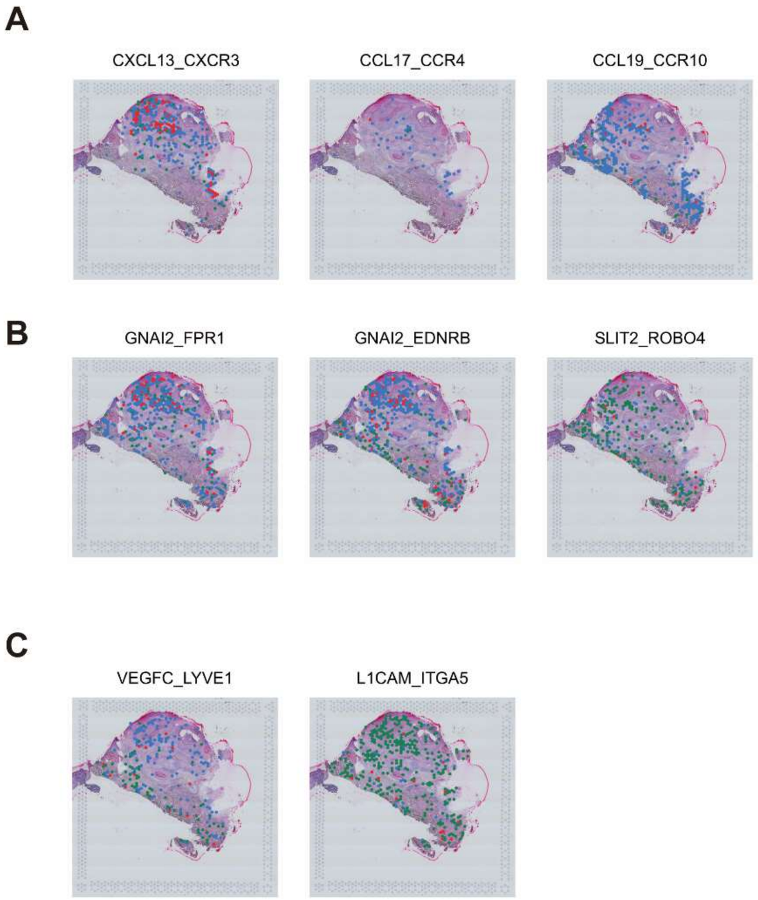

Supplementary Figure 5. Spatial mapping and colocalization of ligand-receptor pairs

Colocalization of ligand-receptor pairs within HNSC tissue for the low-risk group **(A)**, non-significant group **(B)**, and some in high-risk group **(C).**

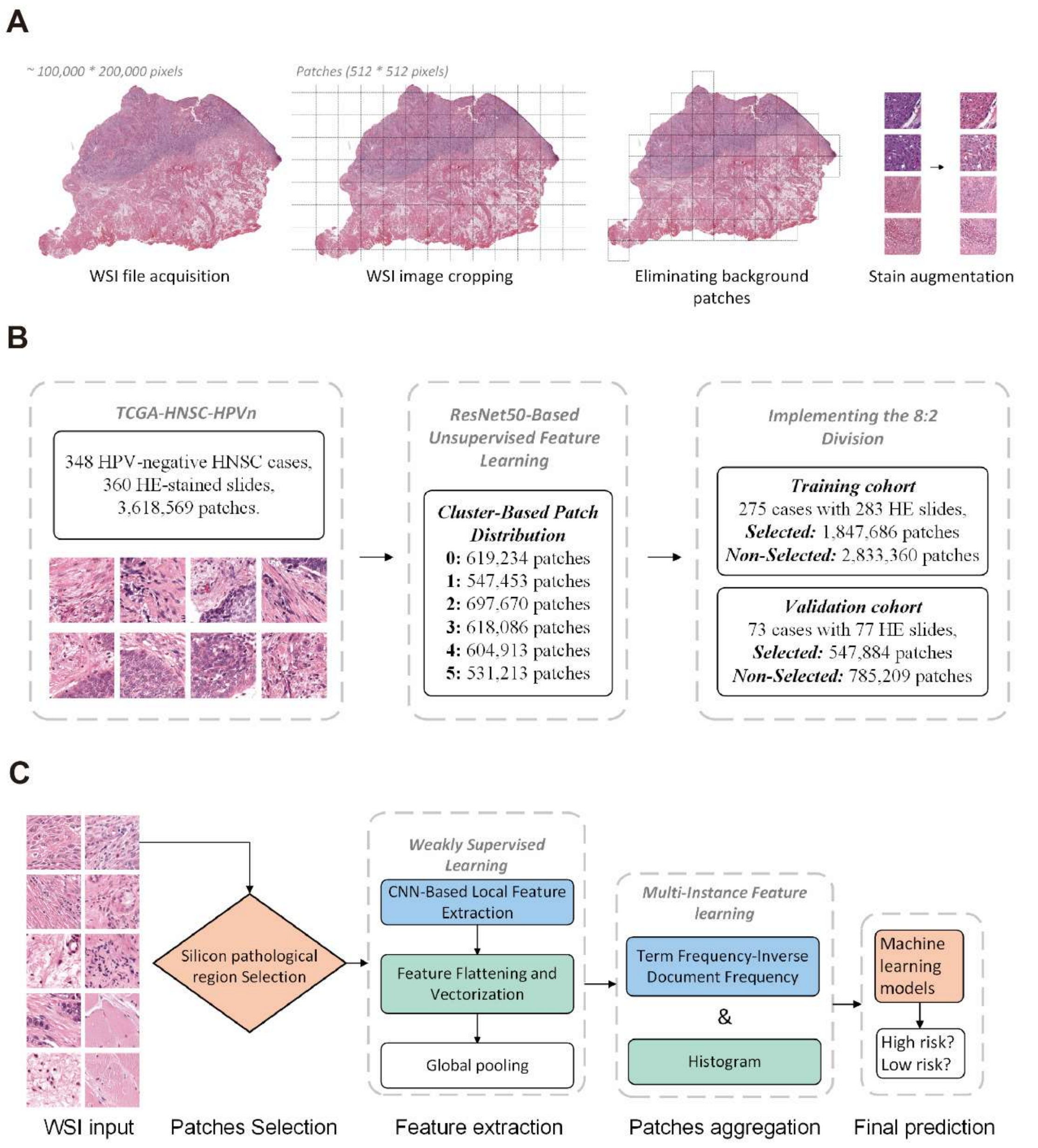

Supplementary Figure 6. Deep learning-based processing of H&E-stained WSI for risk group prediction.

Workflow illustrating the preparation of HE-stained diagnostic pathology WSI. The images are first segmented into 512x512 pixel patches, with background regions removed based on saturation. The Vahadane algorithm is then applied to standardize the colors of the patches **(A)**. This flowchart shows the study pipeline. First, 348 HPV-negative HNSC cases (360 HE-stained slides) yielded 3,618,569 patches. These patches were clustered using a ResNet50-based unsupervised feature approach, then split 8:2 into training (275 cases, 283 slides) and validation (73 cases, 77 slides) cohorts for model development and evaluation **(B)**. Workflow explaining how patches

from specific regions are selected based on Silicon pathological selection. These selected patches are then processed through a convolutional neural network (CNN) to extract deep learning features. Multiple instance learning (MIL) is used to aggregate patch-level features into WSI features. Finally, machine learning models are applied to the WSI features for risk group prediction **(C)**.

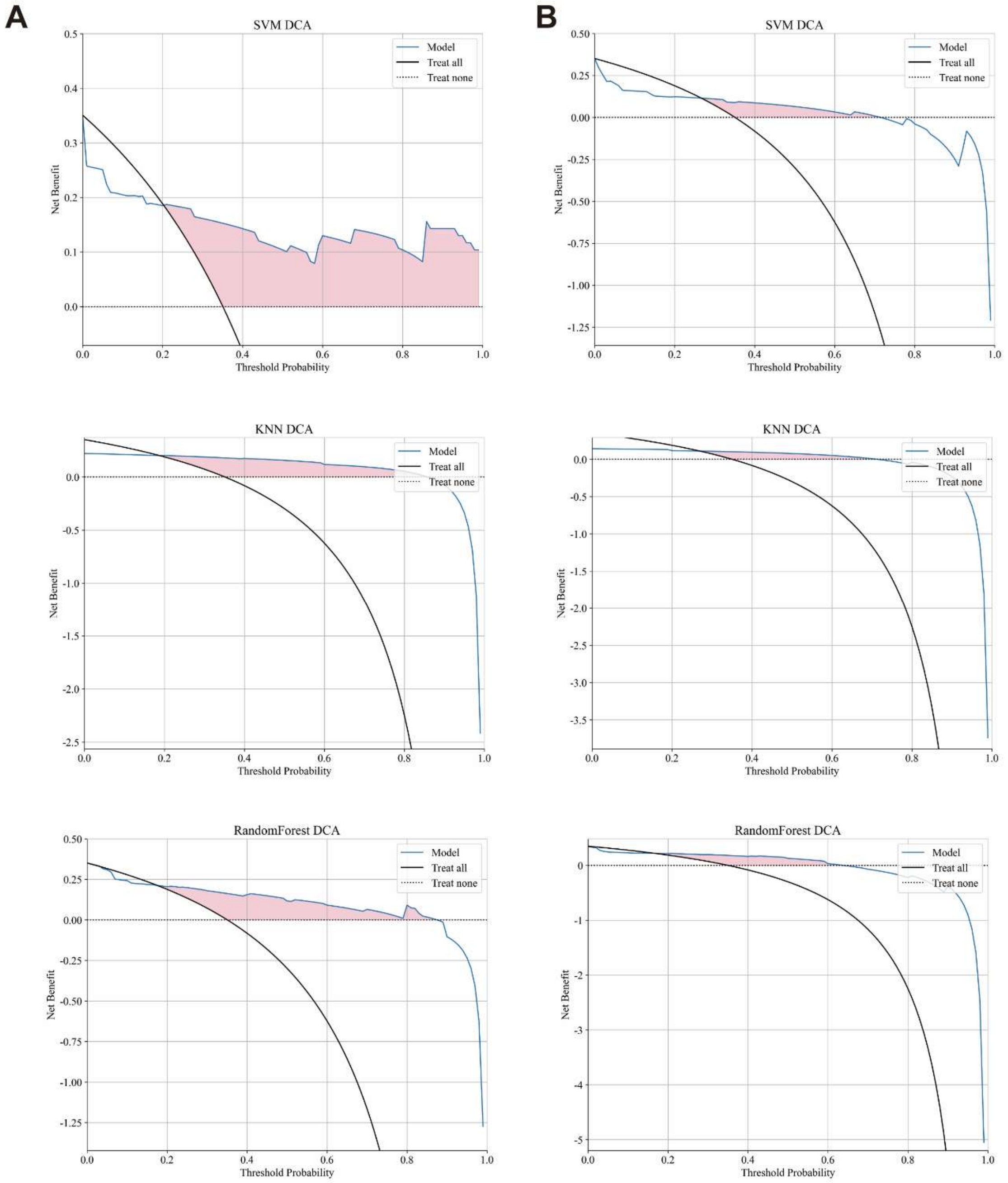

Supplementary Figure 7. Decision curve analysis (DCA) for models with and without Silicon pathological region selection.

DCA plots for SVM, KNN, and Random Forest models are shown with **(A)** and without **(B)** Silicon pathological region selection. The net benefit is displayed as a function of threshold probability across different models.
